# Converting From Laparoscopic Cholecystectomy to Open Cholecystectomy: A Systematic Review of Its Advantages and Reasoning

**DOI:** 10.7759/cureus.64694

**Published:** 2024-07-16

**Authors:** Kapilraj Ravendran, Ahmed Elmoraly, Eirini Kagiosi, Casey S Henry, Jenisa M Joseph, Chloe Kam

**Affiliations:** 1 General Surgery, Cambridge University Hospitals NHS Foundation Trust, Cambridge, GBR; 2 Medicine, Gradscape, London, GBR; 3 General Surgery, East Sussex Healthcare NHS Trust, Hastings, GBR; 4 Medicine and Surgery, Medical University of Sofia, Sofia, BGR; 5 General Surgery, Gradscape, London, GBR; 6 Surgery, Medical University of Sofia, Sofia, BGR; 7 Surgery, Gradscape, London, GBR

**Keywords:** complication, laparoscopic cholecystectomy, gallstone cholecystitis, laparoscopic converted to open, cholecystectomy

## Abstract

Cholecystectomy is the standard treatment for symptomatic cholelithiasis and asymptomatic impending biliary obstruction, which is typically carried out laparoscopically. However, difficult gallbladders, due to distorted anatomy or increased risk of bleeding, can necessitate conversion to open surgery. This systematic review evaluates the advantages, disadvantages, complications, and outcomes of laparoscopic versus converted open cholecystectomy.

We screened articles published from 2011 to 2024 by utilizing advanced filters of PubMed, Cochrane, and Scholar databases. Exclusion criteria included non-English language articles, duplicates, and animal studies. After analyzing relevant articles, 31 articles were included in this study. The total number of participants who underwent laparoscopic procedures was 28,054, of which 5,847 were converted from laparoscopic to open procedures. Conversions were primarily due to bleeding, adhesions, and obscured anatomy, with bile leakage being the most common short-term complication. Converted cases showed higher rates of long-term complications, increased hospital stays, and higher morbidity and mortality. Laparoscopic cholecystectomy remains safe and effective, but identifying high-risk patients for conversion is important. Preoperative identification of high-risk patients and recognizing predictive factors for conversion can enhance surgical outcomes and cost-effectiveness. While laparoscopic cholecystectomy is generally preferred, timely conversion to open surgery is essential for patient safety.

## Introduction and background

Gallstone disease is a major health problem in Western countries, affecting up to 15% of the population. The incidence of gallstones increases with age and is more common in women, affecting 20% of women and 5% of men between the ages of 50 and 65 years [1,2].

Cholecystectomy is the recommended treatment for symptomatic gallstone disease [1,3,4] and is a procedure performed very frequently, with 300,000 laparoscopic cholecystectomy procedures performed annually in the United States [1]. Complications of cholecystectomy include bleeding, such as from the highly vascular liver bed, infection, bile leak, or, of particular concern, iatrogenic damage to the remainder of the biliary tree, potentially requiring major reconstructive surgery [1,5].

The term "difficult gallbladder" is used to describe those gallbladders where an underlying pathology increases the challenge of the procedure, due to either distorted anatomy or increased bleeding risk [3,5]. The incidence of a difficult gallbladder is up to 16% [5,6] and is associated with a higher risk of surgical complications, in particular bile duct injury (BDI) [2,5,7].

There are several techniques used to reduce the risks associated with a difficult gallbladder during laparoscopic cholecystectomy. These include conversion to open cholecystectomy, cholecystostomy drain alone, fundus-first approach, or subtotal cholecystectomy, which may or may not involve removal of the posterior gallbladder wall, closure of the cystic duct opening, or reconstitution of a gallbladder pouch [2,5,7-9].

Data from the literature indicate that, for a variety of reasons, 2 to 15% of laparoscopic cholecystectomies end up as open surgeries [10]. Peritoneal adhesions and inflammatory gallbladder infiltration are the most frequent causes [10]. Converted cases are linked to a higher rate of readmission within 30 days, a higher number of infectious and other postoperative complications, and an increased risk of further procedures [10]. Furthermore, patients in this cohort experience increased morbidity and mortality rates as well as lengthier postoperative stays following conversion from laparoscopic to open surgery [10].

The aim of this systematic review was to analyze the literature and compare converting laparoscopic to open cholecystectomy to review the advantages, disadvantages, complications, and outcomes of both and identify any particular patient groups that convey additional benefits from each type.

## Review

Methodology

Search Strategy and Inclusion Criteria

This study is a systematic review of reviews that evaluated the benefits and rationale for conversion from laparoscopic to open cholecystectomy. This review was carried out in compliance with the PRISMA (Preferred Reporting Items for Systematic Reviews and Meta-analyses) guidelines [11] and the protocol that was jointly developed and approved by all authors. Bias risk was evaluated, and differences in opinion about bias and how to interpret the data were settled through discussions leading to a consensus.

Using the following set of keywords, a literature search was conducted through MEDLINE (accessed through PubMed), Cochrane (Cochrane Database of Systematic Reviews, Cochrane Central Register of Controlled Trials, Cochrane Methodology Register, Database of Abstracts of Reviews of Effects, National Health Service Economic Evaluation Database), and Google Scholar: “cholecystectomy” OR “cholelithiasis” AND “laparoscopic cholecystectomy” AND “laparoscopic converted to open cholecystectomy” combined with Medical Subject Heading “laparoscopic surgery”.

The search was restricted to publications in English.

Study Selection

The systematic review inclusion criteria were reasons for converting from laparoscopic to open cholecystectomy, gallbladder thickness for conversion, outcomes of converting to open cholecystectomy, and hospital stay. Additionally, screening was done for subhepatic fluid collection, bile leak, and short- and long-term complications as secondary outcomes. Studies with non-English writing and those whose complete texts were not accessible online met the exclusion criteria. Our review contained only case reports, case studies, prospective and retrospective research, and randomized controlled trials. Editorials and systematic reviews were not included.

Three researchers' titles and abstracts served as the basis for the initial screening (E.K., C.S.H., and C.K.). Two investigators (K.R. and J.M.M.) reconciled their differences regarding bias assessment and result interpretation. Abstracts with incomplete information were retrieved for analysis of the full text. The eligibility of full-text articles was then independently assessed by the same investigators. Consensus talks were used to settle disagreements regarding the evaluation of bias and the interpretation of the findings. After review, the full texts of the remaining articles were obtained and removed if they were found to be unrelated to the review topic.

Data Extraction and Quality Assessment

Data extraction was carried out by four researchers (E.G., C.S.H., J.M.M., and C.K.) using standardized criteria, and the findings were examined by two senior researchers (K.R. and A.E.). The following information was extracted: journal, year of publication, databases searched, duration, number of studies, total number of patients and countries, study design, results, primary findings, primary limitations, and implications: opportunities and challenges.

The included articles were examined, and information about the findings of our study was extracted. The PRISMA guidelines were followed in this systematic review [11].

Results

**Figure 1:**
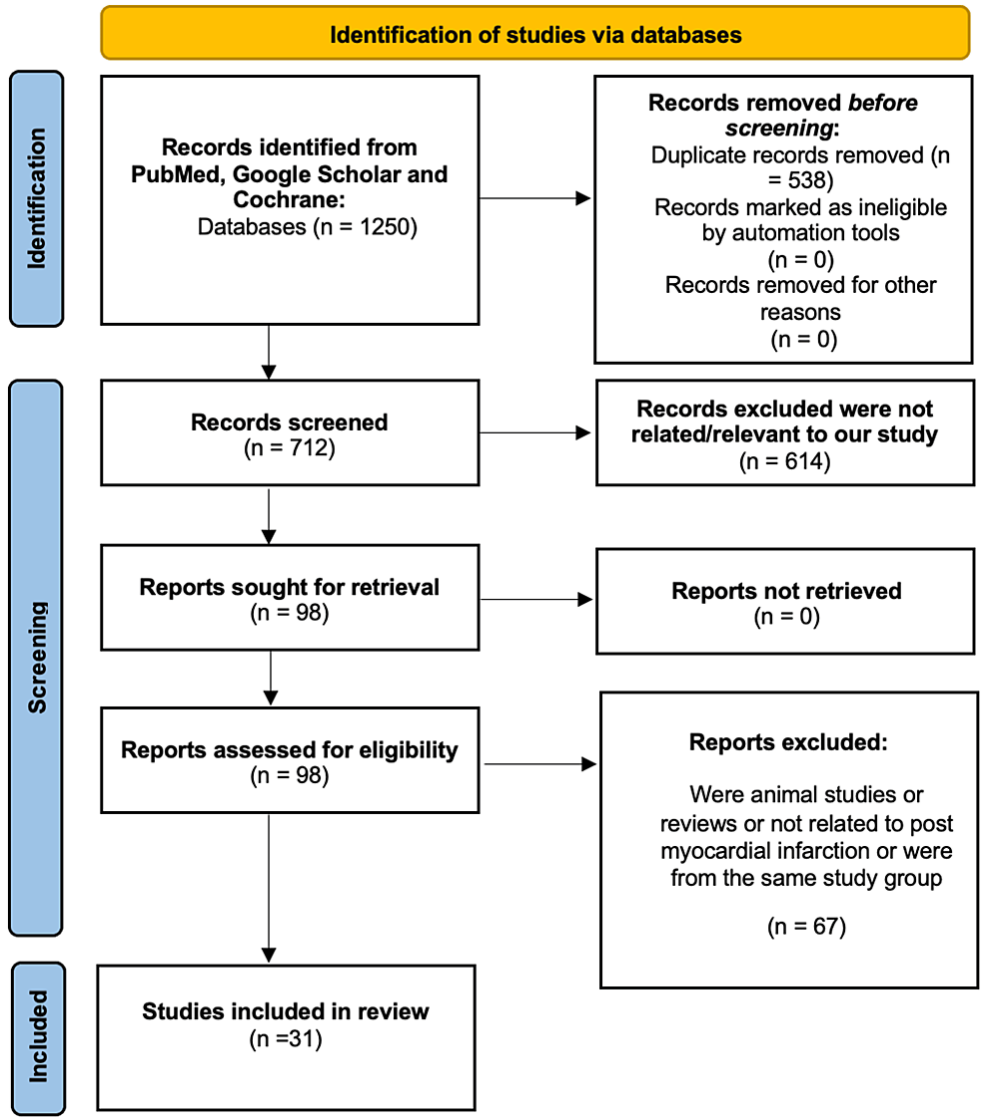
PRISMA flow diagram demonstrating the literature selection strategy. PRISMA: Preferred Reporting Items for Systematic Reviews and Meta-Analyses.

The initial database search yielded 1250 articles, of which 538 were excluded due to duplication. None of the remaining were removed upon applying the exclusion criteria. Abstracts were screened, and 614 articles were removed on the grounds of irrelevance to the review topic or being published before 2011. Full-text copies of 98 articles were obtained, with none being unavailable. A further 67 were removed after review for being irrelevant to the review topic. A total of 31 articles were included in the final review. A PRISMA-style diagram is shown in Figure 1 to demonstrate the selection of literature [11].

The total number of participants who underwent laparoscopic procedures was 28,054, while those who had switched from laparoscopic to open procedures were 5,847 [12-42]. Bleeding, adhesions, and obscured anatomy were some of the most common reasons for conversion in those patients, with bile leakage being the most common short-term complication. Long-term complications were presented more in converted patients, as well as longer hospital stays. Overall, the laparoscopic procedure is still considered safe and reliable with promising results in the field of cholecystectomy [12-42].

Table 1 summarizes our findings from the literature included in our inclusion criteria for laparoscopic converted to open cholecystectomy.

**Table 1:** Studies analyzed in the review with a focus on reasoning, complications, gall bladder thickness, length of stay, and bile leak Lap: laparoscopic, CBD: common bile duct injury, N/A: Not available or not applicable.

Source	Type of study	Participants/personnel	Histological findings for Lap to open	Gallbladder thickness for Lap to open	Reason for conversion to open	Short-term complications	Long-term complications	Outcome/conclusions	Length of hospital stay	Subhepatic fluid collection	Bile leak
Ciftci et al. [12]	Case report	302 for Lap; 10.5% (n = 32) Lap converted to open	43.75% (n = 14) gangrenous; 28.1% (n = 9) phlegmonous	5-9 mm: 28.1% (n = 9) cases; >10 mm: 71.9% (n = 23) cases	18.75% (n = 6) bleeding; 9.4% (n = 3) suspected choledocholithiasis; 6.2% (n = 2) bile duct injury; 6.2% (n = 2) – technical difficulty	N/A	Surgery site infections: Lap 2.2% (n = 6); Lap to open 12.75% (n = 4)	Male gender; gangrenous cholecystitis; and a wall of >1 cm is associated with a higher risk of conversion	Lap: 1±0.8 days; Lap to open: 1±2.5 days	Lap: 0.4% (n = 1); Lap to open: 9.4% (n = 3)	Lap: 0.74% (n = 2); Lap to open: 3.1% (n = 1)
							Adhesion: Lap 0% (n = 0); Lap to open 59.4% (n = 19)				
							Uncontrolled bleeding: Lap 0% (n = 0); Lap to open 18.75% (n = 6)				
							Atelectasis: Lap 1.1% (n = 3); Lap to open 15.6% (n = 5)				
							Choledocholithiasis: Lap 0% (n = 0); Lap to open 9.4% (n = 3)				
Shrestha et al. [13]	Case report	348 patients for Lap; 6.9 % converted from Lap to open (n = 24)	N/A	N/A	41.6% (n = 10) dense inflammatory adhesions and poor display of Calot’s anatomy; 29.1% (n = 7) acute inflammation; 12.5% (n = 3) obscure anatomy; 8.3% (n = 2) bleeding; 4.16 % (n = 1) CBD injury; 4.16% (n = 1) GB empyema	Lap to open: 0.28% (n = 1) bile duct injury; 0.28% (n = 1) diffuse bleeding in gallbladder fossa	N/A	Lap procedure is considered more safe, reliable and promising	3.8 days, for all participants	N/A	Lap to open: 0.86% (n = 3)
Taki-Eldin et al. [14]	Case report	492 patients for Lap; 4.9% converted from Lap to open (n = 24)	N/A	N/A	0.8% (n = 4) obscure anatomy; 2.2% (n = 11) difficult dissection in Calot’s triangle due to acutely inflamed edematous gallbladder or adhesions; 1.8 % (n = 9) excessive uncontrolled bleeding interfering with clear visibility	Lap: 1.4% (n = 7) tear in common bile duct	Lap: 1.8% (n = 9) post-incisional hernia; 4.3% (n = 21) wound infections; 1.6% (n = 8) collections in Morrison’s pouch	N/A	2.6+-1.5 days for all		Lap: 2.4% (n = 12)
Suzuki et al. [15]	Case report	25 participants for Lap; 32% (n = 8) converted from Lap to open; 50% (n = 4) were planned for open	N/A	N/A	75% (n = 6) adhesions; 12.5% (n = 1) bleeding; 12.5% (n = 1) pneumoperitoneum:	Lap: 8% (n = 2) postoperative bile duct obstruction; Lap to open: 12.5% (n = 1) hemoperitoneum	No mortality	High-risk patients for acute cholecystitis., have a higher rate of conversion and hepatobiliary morbidity rates	8.8 days for all		Lap: 8% (n = 2)
Haider et al. [16]	Case report	202 participants for Lap; 1.5% (n = 3) converted from Lap to open	N/A	N/A	100% (n = 3) obscured anatomy in Calot’s triangle and GB empyema:	Lap: 4% (n = 8) port site infection; 2.5% (n = 5) chest infections; 0.5% (n = 1) myocardial infarction	N/A	Lap is a safe procedure with the advantages of decreased wound infections, less pain, and early recovery	Lap to open: 2 ± 0.7 days	N/A	N/A
Amreek et al. [17]	Case report	855 participants for Lap; 3.6% (n = 31) converted from Lap to open	N/A	>8 mm	N/A	Intra-abdominal infections: Lap 0.7% (n = 6); Lap to open 0% (n = 0)	Lap converted to open: 6.5% (n = 2) deaths	Both intra- and post-operative complications were higher in conversion patients	N/A	N/A	Lap: 0.4% (n = 3); Lap converted to open: 3.2% (n = 1)
						Surgical site infections: Lap 2.4% (n = 20); Lap to open 9.7% (n = 3)					
						Biliary peritonitis: Lap 1% (n = 8); Lap to open 6.5% (n = 2)					
						Fecal peritonitis: Lap 0.5% (n = 4); Lap to open 3.2% (n = 1)					
						CBD stones: Lap 0.1% (n = 1); Lap to open 0% (n = 0)					
						Jaundice: Lap 1.1% (n = 9); Lap to open 6.5% (n = 2)					
Sapmaz et al. [18]	Case report	1294 participants for lap; 3.2% (n = 41) Lap converted to open	N/A	>3 mm	14% (n = 6) adhesions; 9.7% (n = 4) cystic duct injury; 9.7% (n = 4) CBD injury; 9.7% (n = 4) CBD stone; 9.7% (n = 4) dissection difficulty; 9.7% (n = 4) stomach-bowel injury; 4.8% (n = 2) adhesions from previous surgery; 4.8% (n = 2) anatomic variations; 4.8% major (n = 2) abdominal vascular injury; 4.8 % (n = 2) stone loss	2.4% (n = 1) that had cystic duct injury was reoperated 2 days later	N/A	acute cholecystitis appears to be the most significant factor increasing the rate of conversion to open surgery during lap procedures	Lap converted to open: 1.2 days	N/A	Lap: 17% (n = 7)
Fletcher et al. [19]	Case report	547 participants for Lap; 6% (n = 33) Lap converted to open	N/A	N/A	15% (n = 5) major bile duct injury; 18% (n = 6) organ injuries; 70% (n = 23) surgeons’ decision	Lap: 3.8% (n = 21) infection; 1.1% (n = 6) retained stone; 0.9% (n = 5) bleeding; 0.9% (n = 5) major biliary injury	N/A	Lap: Mortality rate was low, 0.004%, from septic shock	Not reported	N/A	Lap: 3.8% (n = 21)
Geraedts et al. [20]	Case report	1553 patients for Lap; 2.7% (n = 42) Lap converted to open	N/A	N/A	hostile abdomen 31% (n = 13); perforation of the gallbladder 26% (n = 11); insufficient overview of the hilar structures 2.4% (n = 1); suspected injury of the common bile duct 4.8% (n = 2)	Lap: 1.1% (n = 17) retained stone 0.06% (n = 1) bleeding	Lap: 0.3% (n = 5) pancreatitis; 2.9% (n = 45) site infections; 1.2% (n = 19) abscess	Lap: 0.3% (n = 5) patients died	N/A	N/A	Lap: 1% n = 16
Ozsan et al. [21]	Case report	149 participants for Lap; 2.69% (n = 4) Lap converted to open	N/A	>5 mm	Severe peritoneal adhesions: 75% (n = 3); dense adhesions around gallbladder: 25% (n = 1)	N/A	N/A	Lap cholecystectomy with continuous pressurized irrigation and dissection technique seems to be an effective and reliable procedure with low complication and conversion rates	Lap to open: 3.93 days	Lap: 2% (n = 3)	N/A
Feng et al. [22]	Case series	48 patients for Lap; 16.7% (n = 8) Lap converted to open	N/A	>3 mm	Severe pericholecystic adhesions and suspicion for GB carcinoma: 100% (n = 8)	Lap: 6.6% (n = 3) wound infections; 15.5% (n = 7) abdominal infections; 4.4% (n = 2) pneumonia	Lap: 2.2% (n = 1) Deep Venous Thrombosis	N/A	4.3 days in all included in series	N/A	N/A
Özdemir et al. [23]	Retrospective analysis	921 patients for Lap; 3% (n = 28) Lap converted to open	N/A	>3 mm in 68% (n = 19); <3 mm in 32% (n = 9)	Adhesion due to inflammation: 53.6% (n = 15); inability to see the hilum: 21.4 % (n = 6); adhesions due to previous operations: 14.3 % (n = 4); hemorrhage: 7.1% (n = 2); organ injury: 3.6% (n = 1);	N/A	N/A	The most prevalent reason for conversion from Lap to open surgery was adhesion owing to inflammation	Lap: 1.8 days Lap converted to open: 7 days	N/A	N/A
Tay et al. [24]	Retrospective analysis	153 patients for Lap; 16.3% (n = 25) Lap converted to open	N/A	N/A	N/A	N/A	N/A	There was no 30-day or operation-related mortality	Median stay for all patients: 4 days	N/A	N/A
Yilmaz et al. [25]	Retrospective study	320 patients for Lap; 12.2% (n = 39) Lap converted to open	N/A	N/A	Adhesion: 89.7% (n = 35); bleeding: 10.3% (n = 4)	N/A	N/A	N/A	Median hospital stays for all patients: 1.70 ± 1.43 days	N/A	N/A
Sushma et al. [26]	Retrospective study	85 patients for Lap; 6% (n = 5) Lap converted to open	N/A	N/A	Frozen Calot’s triangle: 20% (n = 1); gangrenous gallbladder:20% (n = 1); Dense adhesions: 20% (n = 1); bleeding that obscured the vision:20% (n = 1); Choledochal cyst: 20 % (n = 1)	N/A	N/A	The most common cause for conversion was difficult dissection of Calot’s triangle	Average hospital stays for all patients: 3 days	N/A	N/A
Verma et al. [27]	Retrospective cohort study	430 patients for Lap; 3.3% (n = 14) Lap converted to open	N/A	N/A	Unclear Calot’s triangle anatomy: 27.2% (n = 3); bleeding from gallbladder bed 18%: (n = 2); dense adhesion in gallbladder: 9% (n = 1); anesthesia problem: 9% (n = 1)	N/A	N/A	Preoperative difficult Lap cholecystectomy can be predicted and modifications of the steps of four-port cholecystectomy can be done to minimize the conversion rate	Lap: 2.3+0.8 days; Lap Converted to open: 6.9+1.8 days	N/A	N/A
Oymaci et al. [28]	Retrospective analysis	165 patients for Lap; 28% (n = 46) Lap converted to open	N/A	>4 mm in 93% (n = 43)	Inability to define anatomy: 45.7% (n = 21); dense adhesions of the gallbladder: 39% (n = 18); hemorrhage: 6.5% (n = 3); Mirizzi syndrome: 6.5% (n = 3)	N/A	N/A	Male sex, blood leukocyte, and raised glucose, and amylase have emerged as the effective factors for conversion cholecystectomy	Lap: 1.75 ±0.87 days; Lap Converted to open: 3.39 ± 2.38 days	Lap:0.8% (n = 1)	Lap:1.7% (n = 2); Lap converted to open: 2.2% (n = 1)
Saeed et al. [29]	Prospective observational study	100 patients for Lap; 7% (n = 7) Lap converted to open	N/A	N/A	dense adhesions at the Calot’s triangle: 71.43%) (n = 5); obscure anatomy 42.86% (n = 3)	N/A	N/A	adhesions at the Calot’s triangle and obscure anatomy are the two main Intra-operative factors responsible for conversion	Lap: 2.29 days; Lap converted to open: 5.57 days	N/A	N/A
Al Masri et al. [30]	Retrospective study	4668 patients for Lap; 1% (n = 48) Lap converted to open	N/A	Wall thickness >4 mm lap to open: 41.7% (n = 20)	difficult anatomy; obscured view; severe adhesions; significant inflammation	N/A	Lap converted to open: 2% (n = 1) death	Advanced age, male gender, significant comorbidities, and history of prior laparotomies have a high risk of conversion	Lap: 2 ± 4 days; Lap converted to open: 9.85 ± 7.13 days	N/A	Lap: 0.02% (n = 1)
Ekici et al. [31]	Retrospective study	173 patients for Lap; 8% (n = 14) Lap converted to open	N/A	N/A	N/A	Lap bleeding from trocar insertion site: 1.25% (n = 2); re-operation from postoperative hemorrhage: 0.6% (n = 1);	N/A	A high WBC count (>10,000/mm³) with a conversion rate of 25.0% was seen	Mean hospital stay for all patients: 3.4 days	N/A	N/A
Javaid et al. [32]	Retrospective analysis	250 patients for Lap; 2.4% (n = 6) Lap converted to open	N/A	N/A	Dense adhesions in Calot’s triangle: 50% (n = 3); cholecystoduodenal fistula: 16.6% (n = 1); Previous upper abdominal surgeries: 16.6% (n = 1); Mirizzi’s syndrome: 16.6% (n = 1)	N/A	N/A	Dense adhesions around the Calot’s triangle were the most common cause of conversion	Average hospital stays for all patients: 1-3 days	N/A	N/A
Madhusudhan et al. [33]	Retrospective observational study	200 patients for Lap; 8% (n = 16) Lap converted to open	N/A	Thickened GB wall: 32 and only 50% (n = 16) underwent conversion	Dense adhesions 62.5% (n = 10); bleeding from Calot's triangle or the gallbladder bed: 25.0% (n = 2); intraoperative injuries like common bile duct injury or pyocele: 12.5% (n = 1)	Lap Common bile duct injury/ pyocele: 12.5% (n = 25)	N/A	The main cause of conversion from Lap cholecystectomy to open was difficult anatomy secondary to dense adhesions followed by bleeding	N/A	N/A	N/A
Kala et al. [34]	Retrospective analysis	8347 patients Lap; 1% (n = 82) Lap converted to open	Scleroatrophic gallbladder: 1.2% (n = 1); Gangrenous cholecystitis: 1.2% (n = 1)	N/A	Acute cholecystitis: 2.5% (n = 2); dense adhesions at triangle of Calot:2.5% (n = 2); Mirizzi syndrome 62.%) (n = 51); empyema: 7.3% (n = 6); Cirrhosis:7.3% (n = 6); Acute biliary pancreatitis: 4.76% (n = 4); Cholecysto-enteric fistula: 8.33% (n = 8)	Lap CBD injury: <1 % (n = 5); -post-op fever 0.2% (n = 18); hemorrhage:<1% (n = 6)	N/A	Conversion was needed in n = 3.75% of patients. This shows that Lap can be performed safely with fewer conversions and fewer complications	Lap to open: 4.8 days; Lap: 2.7 days	N/A	N/A
Awan et al. [35]	Retrospective observational study	450 patients for Lap; 5.8% (n = 26) Lap converted to open	N/A	<2 cm: 19.2% (n = 5) cases; 2-5 cm: 61.5% (n = 16); cases >5 cm: 19.2% (n = 5) cases	Dense adhesions: 46% (n = 12); obscure anatomy at Calot's triangle: 23% (n = 6); bleeding: 19.2% (n = 5); Visceral injury: 3.8% (n = 1); instrument failure: 3.8% (n = 1); CBD injury: 3.8% (n = 1)	Lap intra-operative bleeding: 1% (n = 5); CBD injury: 0.2% (n = 1)	N/A	Lap is a more effective and safe procedure; however, conversion to open surgery is inevitable in some difficult cases and is required for the safety of the patients	N/A	N/A	N/A
Kumar et al. [36]	Prospective study	112 patients for Lap; 10.7% (n = 12) Lap converted to open	N/A	N/A	Difficult anatomy due to dense adhesions of Calot’s triangle: 50% (n = 6); Anatomical variation: 8.3% (n = 1); Bleeding from Calot’s triangle:16.6% (n = 2); Injury to right gastric artery: 8.3% (n = 1); Common bile duct injury: 16.6% (n = 2)	N/A	N/A	Lap has better results compared to conversion to open. Hospital stay for Lap was n = 3.37, which is shorter compared to open	Lap: 3.37 days; Lap to open: 5.75 days	N/A	N/A
Chandramouli et al. [37]	Prospective study	28 patients for Lap; 7.1% (n = 2) Lap converted to open	N/A	N/A	Acute cholecystitis:100% (n = 2)	N/A	N/A	The most common reason for conversion was acute cholecystitis. Hospital stay was longer in the converted group compared to the lap	N/A	N/A	N/A
Hirohata et al. [38]	Retrospective analysis	395 patients for Lap; 8.4% (n = 33) Lap converted to open	N/A	N/A	N/A	Lap Bile leakage: 0.8% (n = 3); biliary injury: 0.2% (n = 1)	N/A	Lap to open:6% (n = 2) deaths	Lap: 7 days; Lap to open: 12 days	N/A	N/A
Ramirez-Giraldo et al. [39]	Retrospective observational cohort study	144 patients for Lap; 13.2% (n = 19) Lap converted to open	N/A	N/A	N/A	Lap bile duct injury: 6.4% (n = 8)	N/A	Lap converted to open: 36.8% (n = 7) deaths	10 days for all patients	N/A	N/A
Özkardeş et al. [40]	Randomized Controlled Trial	60 patients for Lap; 6.6% (n = 4) Lap converted to open	N/A	N/A	N/A	Lap Intraoperative bleeding: 5% (n = 3)	Lap converted to open Lung infection: 1.8% (n = 1)	N/A	5.2 ± 1.40 days for all patients	N/A	N/A
Al-Dhahiry et al. [41]	Prospective study	128 patients for Lap; 5.5% (n = 7) Lap converted to open	N/A	N/A	Adhesion due to previous upper midline laparotomy: 14.3% (n = 1); unclear anatomy at Calot's triangle:28.6% (n = 2); cholecystoduodenal fistula:14.3% (n = 1); Mirizzi’s syndrome: 14.3% (n = 1); incidental stone in CBD: 14.3% (n = 1); impacted stone in cystic duct: 14.3% (n = 1)	N/A	N/A	Unclear anatomy was the most common cause for conversion in this study	Lap: 1-4 days; Lap to open: 7 days	N/A	N/A
Genc et al. [42]	Retrospective analysis	5382 patients for Lap; 5164 Lap converted to open	N/A	N/A	Adhesions caused by tissue inflammation: 2% (n = 97); fibrosis of Calot's triangle: 0.2% (n = 12)	N/A	N/A	Male gender was found to be the only statistically significant risk factor for conversion in our series. LC can be safely performed with a conversion rate of less than 5% in all patient groups	N/A	N/A	N/A

Discussion

Strong, repeatable metrics are clearly needed to enable patients with cholecystitis to understand the severity of their illness. Determining the gallbladder's condition at surgery and the severity of any cholecystitis will make reporting more standardized, enhance pathways, and better manage risk-adjusted outcomes [43]. Remarkably, the first open cholecystectomy was reported by Carl Langenbuch in 1882, and the first laparoscopic cholecystectomy was reported by Muhe in 1985 [43]. Grading the severity of cholecystitis has only recently come to more attention [43]. It is now widely acknowledged that more understanding of the heterogeneity of cholecystitis and variation in outcome is required [43]. Hanna et al. and Nassar et al. published basic difficulty scales for cholecystectomy in the 1980s and 1990s [43]. In 2015, we identified 16 published gallbladder grading systems when we reported the G10 operative scoring system [43]. There have been several reported numbers since then [43]. Shifts in the paradigm for managing biliary disease complicate the variability of operative findings [43].

Grading systems have identified risk factors for both prolonged surgery and increased need for conversion [43]. Wakabayashi et al. identified 19 operative risk factors potentially contributing to conversion [43]. As surgeons, we know that there are unique variable technical difficulties encountered during cholecystectomy and these are fundamentally related to the access, adhesion density and vascularity, and the thickness, friability, weight, and thickness of the gallbladder [43]. Recently, Wakabayashi et al., as part of the Tokyo 2018 guidelines, suggested 25 operative findings with scores that may affect the technical difficulty of cholecystectomy [43].

Determining preoperative patient-related variables and predicting the necessity of switching from laparoscopic to open cholecystectomy surgery can assist in identifying high-risk patients and redefining the surgical approach for this subset of patients [10]. Additionally, by using these predictive conversion factors, gallstone treatment can become more cost-effective and safer for patients [10].

A reason for converting from laparoscopic cholecystectomy to open cholecystectomy is the surgical procedure's time of day, which can be a statistically significant variable [10]. This is particularly applicable to operations carried out when the hospital ward is not staffed by a full complement of skilled and knowledgeable surgeons [10]. Another factor contributing to the ineffectiveness of surgical procedures is the decline in surgeons' psychomotor performance, which happens gradually over the course of a workday and results in a decrease in efficiency [10]. Acute cholecystitis, choledocholithiasis, emergency surgery, diabetes, hypertension, heart disease, neurological disease, and, to a lesser extent, anatomical uncertainty are additional potential risk factors that are statistically significant for an unplanned laparotomy [10]. The patient's status following endoscopic retrograde cholangiopancreatography (ERCP), pancreatitis, peritoneal adhesions, and chronic cholecystitis were not statistically significant as potential conversion factors [10].

An analysis was conducted on perioperative factors that impact the likelihood of an unplanned laparotomy occurring either before or during the procedure [10]. Acute cholecystitis, peritoneal adhesions, and chronic cholecystitis are the most significant [10]. A classification of surgical factors, patient-related factors, equipment-related factors, and the importance of the surgeon's experience is also suggested by other researchers examining the factors that led to the switch from laparoscopic to open surgery [10].

Gallbladder wall thickening may be the most sensitive indicator of conversion in laparoscopic cholecystectomy, according to Tosun et al. [44]. Gallbladder wall thickening frequently signifies the existence of either acute or chronic inflammation [44]. The gallbladder triangle's anatomical relationship is frequently unclear due to chronic inflammation and/or acute inflammatory edema, and this can even result in "frozen" adhesion, which makes surgery more challenging [43]. In patients with CBD stones, inflammatory fibrosis of the CBD wall readily results in local thickening when stones are imprisoned there or when they are repeatedly stimulated physically [44].

Impaction of the duodenum stone's medial wall was linked to longer operating times and surgical failure, as demonstrated by Noble et al. [45]. The stones in this area are easily impacted by the inner segment of the duodenum's small lumen and thick wall, making it impossible for the stone removal basket and forceps to penetrate this area along the bile duct wall or open it during the procedure [45].

Strengths and Limitations

The majority of studies included were published within the last 15 years, and many within the last five years, demonstrating the relevance of this topic and the conclusions reached here.

Further high-quality systematic reviews and meta-analyses, particularly including the many studies published in recent years, would be very beneficial in improving the evidence-based guiding practice on the use of converting laparoscopic cholecystectomy to open cholecystectomy.

## Conclusions

Skilled laparoscopic surgeons should advise patients in the high-risk group about the possibility of converting to open surgery and make the appropriate decision when necessary. The surgeon should feel at ease switching from laparoscopic to open cholecystectomy when the dissection is laborious. The metrics of cholecystitis and cholecystectomy must be accepted by the global surgical community. To progress toward better outcomes for our patients with biliary disease, a consensus peri-operative grading or score of gallbladder disease and surgery must be adopted. The main reasons for converting laparoscopic to open cholecystectomy include bleeding, adhesions, and obscured anatomy, as well as gallbladder thickness. Short-term complications included bile leak and prolonged hospital stay. Despite the fact that this study has some limitations, it provides groundwork for future studies, mainly focusing on the reduction in complications.
